# Determinants of life satisfaction in older adults with diabetes in China: a national cross-sectional study

**DOI:** 10.3389/fpubh.2025.1585752

**Published:** 2025-04-22

**Authors:** Xiaobing Xian, Xiaoli Fan, Xiaowei Wei, Xuemei Wang, Yandi Fu, Damin Sun

**Affiliations:** ^1^The Thirteenth People’s Hospital of Chongqing, Chongqing, China; ^2^Chongqing Geriatrics Hospital, Chongqing, China; ^3^College of Public Health, Chongqing Medical University, Chongqing, China; ^4^School of Foreign Languages, Chongqing Medical University, Chongqing, China; ^5^School of Paediatric, Chongqing Medical University, Chongqing, China

**Keywords:** diabetes, older adults, life satisfaction, random forest, CLHLS

## Abstract

**Background:**

The life satisfaction (LS) of individuals among older adults with diabetes should not be neglected. However, current research provides limited insight into the LS of older adults with diabetes in China. Therefore, the primary objective of this study is to assess the current life satisfaction status of older adults with diabetes in China, to delve into the factors influencing it, and to identify the key factors.

**Methods:**

This study selected 1,304 patients with diabetes from the Chinese Longitudinal Healthy Longevity Survey (CLHLS) database for analysis. A multivariate logistic regression model was used to analyze the factors influencing life satisfaction among diabetic patients, and a random forest model was further utilized to rank the importance of significant influencing factors.

**Results:**

30.14% of older adults with diabetes were dissatisfied with their lives. Multivariate Logistic regression analysis shows that self-assessed health status, self-assessed economic status, depressive symptoms, exercise, living arrangements, hearing impairment, and cognitive impairment all significantly affect the life satisfaction of older adults with diabetics. The OR values for self-assessed health and self-assessed economic status are relatively high, patients with fair and poor self-assessed health was 5.03 times and 9.72 times higher risk of life dissatisfaction compared to those with good self-assessed health (fair: OR = 5.03, 95% CI: 3.46–7.31; poor: OR = 9.72, 95% CI: 6.20–15.26). The risk of feeling dissatisfied with life was 7.69 times higher in patients with poor self-assessed economic status than in those with good self-assessed economic status (OR = 7.69, 95%CI: 4.25–13.89). The random forest results showed that the order of importance from highest to lowest was self-assessed health status, self-assessed economic status, depressive symptoms, exercise, living arrangements, hearing impairment, and cognitive impairment.

**Conclusion:**

Our study reveals that the current rate of life satisfaction among older adults with diabetes is significantly high. Therefore, it is essential to implement measures from multiple perspectives for effective prevention and intervention. Among these factors, priority should be given to interventions focusing on economic support and health management, as these measures may serve as crucial protective factors in enhancing the well-being of older adults with diabetes.

## 1 Introduction

With the rapid aging of China’s population, the proportion of older adults individuals suffering from various chronic diseases is also rising. Diabetes, a chronic metabolic disorder (1), has emerged as a significant public health concern due to its high prevalence, extensive complications, and significant impact on the quality of life of those affected (2). Statistics reveal that between 2000 and 2019, the number of patients with diabetes in China surged from 48 million to 92 million (3). This alarming trend not only poses a serious threat to individual health but also places a substantial burden on families and society. Research indicates that diabetes-related complications, such as diabetic nephropathy and neuropathy (4, 5), pose severe health risks and markedly diminish patients’ quality of life (6). A study conducted on older adults in India showed that individuals with severe diabetes symptoms often experience common mental disorders, which can lead to reduced life satisfaction (7). A study carried out in the north-eastern region of Romania similarly demonstrated a low level of life satisfaction among diabetic patients (8). These findings underscore the importance of a deeper understanding of the epidemiological trends and clinical characteristics associated with low life satisfaction among patients with diabetes. Such knowledge could facilitate the early identification of high-risk groups for low life satisfaction, ultimately enhancing the quality of life for those with diabetes.

Among the protective factors for the development of human health, subjective well-being and psychological well-being are recognised as positive psychological characteristics (9). The concept of quality of life is widely recognised as a health-related issue (10), and life satisfaction not only reflects an individual’s subjective assessment of quality of life, but is also a key indicator of psychological well-being and happiness (11). Diabetes, as a chronic disease that requires long-term management and treatment, not only directly affects the patient’s physiological functions but can also trigger a series of psychological issues and social adaptation disorders, thereby having a profound impact on patient’s life satisfaction. Extensive previous studies have reported various factors influencing the LS of older adults individuals with diabetes, such as emotional distress and cognitive function, which have a significant impact on LS (12). Other research findings suggest that limitations in physical function, such as changes in visual ability, pain, and fatigue, significantly reduce LS in patients with diabetes (13). There is no doubt that these meaningful studies have made a significant contribution to improving the life satisfaction of older adults individuals with diabetes. However, the factors influencing life satisfaction in the Chinese older adults with diabetes are subject to further debate. In addition, the research methods in previous studies tended to use traditional Logistic regression model analysis. Logistic regression modelling analysis is mainly used to investigate the relationship between a categorical outcome and multiple influencing factors, and is one of the most commonly used methods to predict outcomes (14). As a machine learning algorithm, the random forest has become an excellent clinical research tool due to its powerful classification ability and easy-to-understand learning mechanism. In recent years, the random forest algorithm has been widely applied in the medical field for disease diagnosis and classification (15), clinical outcome prediction, and estimation of the importance of exposure to pathogenic factors (16, 17). Previous studies have shown that the performance of the RF algorithm is superior to other algorithms, such as support vector machines and neural network methods, in terms of handling missing values, ease of interpretation, and implementation (18). Furthermore, the random forest algorithm is considered highly stable and effective in reducing overfitting and variance (19). The Mean Decrease in Gini coefficient (MDG) refers to the average reduction in Gini impurity for a particular predictor variable in the random forest. MDG has been proven to be sensitive and stable to predictor variables measured on different scales (20), and therefore, using MDG to evaluate the contribution of each explanatory variable to the heterogeneity of observed outcomes at various nodes of the tree will be reliable.

In summary, we use data from the 65 and older population released by the Chinese Longitudinal Healthy Longevity Survey (CLHLS) in 2018 to comprehensively analyze the factors influencing life satisfaction among patients with diabetes from four dimensions: individual characteristics, psychological and behavioral characteristics, interpersonal relationships, and living and working conditions. We further rank the importance of these factors employing the Random Forest algorithm. The findings provide significant reference value for interventions to improve life satisfaction among older adults diabetics and promote overall mental health in the older adults.

## 2 Method

### 2.1 Data source

The Chinese Longitudinal Healthy Longevity Survey (CLHLS) is the earliest and longest-running national social science survey in China (21). The project is organized by the Center for Healthy Aging and Development Studies/National School of Development at Peking University. It is a follow-up survey of the older adults conducted in 23 provinces, autonomous regions, and municipalities directly under the central government of China. Data from individuals aged 65 and above were collected using a multi-stage cluster sampling method. Data collection was implemented through a structured questionnaire survey, comprising two distinct components: questionnaires for living participants and proxy questionnaires for deceased older adults individuals completed by family members. The survey instrument for living respondents encompassed multiple dimensions, including demographic characteristics and family structure, socioeconomic status and economic resources, self-rated health status and quality of life measures, cognitive functioning assessments, personality traits and psychological characteristics, activities of daily living and instrumental activities of daily living, lifestyle factors, caregiving arrangements, and disease management with healthcare expenditure burden. The most recent wave of the longitudinal survey (2017–2018) successfully collected data from 15,874 individuals aged 65 years and older. The CLHLS project was approved by the Biomedical Ethics Committee of Peking University (IRB00001052-13074), and all participants signed a written informed consent form.

The minimum sample size required for the study was estimated by the researchers using the overall rate. A review of the extant literature revealed an absence of reports on the rate of life dissatisfaction among older adults with diabetes. Consequently, the prevalence rate of 50% was utilised to calculate the minimum sample size required for the study (22, 23). Additionally, the tolerance error was specified as 0.1 of the prevalence rate, which was incorporated into the equation $N=\frac{{U^{2}}_{\alpha /2}p\left(1-p\right)}{\delta^{2}}=\frac{1.96^{2}*0.5*\left(1-0.5\right)}{{\left[0.5*0.1\right]}^{2}}$ =384.16 ≈ 385. The minimum sample size required for the final study is 385 (Where α is the significance level, taking α = 0.05., U_α/2_ = 1.96, *p* is the prevalence rate, *δ* is the allowable error).

**Figure 1:**
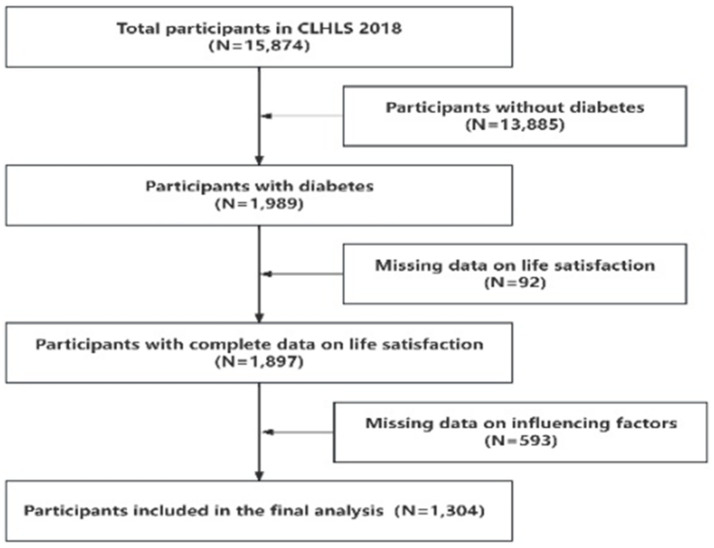
Flowchart of data cleaning.

In 2017–2018, a total of 15,874 older adults individuals were visited. Participants were asked, “Has a doctor ever told you that you have diabetes?” Those who answered “yes” were classified as having diabetes (Includes Type I and Type II) (24). Considering the integrity of the information and avoiding the occurrence of bias, we further excluded the participants who were missing or abnormal in the variable information of life satisfaction and influencing factors, and finally included 1,304 valid samples. The specific data cleaning process flowchart is shown in Figure 1.

### 2.2 Life satisfaction

Life satisfaction (LS) is assessed through a single question: Evaluate your own life as a whole, how satisfied are you with your life? The answer to the question has five options: (1) Completely satisfied; (2) Very satisfied; (3) Fairly satisfied; (4) Not very satisfied; (5) Not at all satisfied. Responses (1), (2), and (3) are classified as “satisfied,” and the remaining options are classified as “dissatisfied.” Previous research has shown the reliability and validity of measuring life satisfaction based on a single question (25).

### 2.3 Influencing factors

Based on previous research experiences (26, 27), we defined 27 potential influencing factors from the CLHLS, which were categorized into four dimensions: individual characteristics, psychological and behavioral characteristics, interpersonal relationships, and living and working conditions.

In terms of individual characteristics, we considered age, gender, education level, residence, BMI, abdominal obesity, hypertension, heart disease, visual impairment, hearing impairment, and based on the Katz Index, we also assessed activities of daily living (ADL) disabilities to determine the presence of ADL disabilities (28). Cronbach’s alpha coefficient was 0.834 and the Kaiser-Meyer-Olkin (KMO) measure was 0.865.

For psychological and behavioral characteristics, we used the Generalized Anxiety Disorder (GAD-7) scale to assess patients with diabetes’ anxiety symptoms (29), the Cronbach’s alpha of the scale in this study is 0.904, and the KMO is 0.913, the CES-D-10 scale to assess patients with diabetes’ depressive symptoms (30), The Cronbach’s alpha of the scale in this study is 0.750, and the KMO is 0.835 and the Chinese version of the Mini-Mental State Examination (MMSE) to evaluate patients with diabetes’ cognitive function (31), the Cronbach’s alpha of the scale in this study is 0.852, and the KMO is 0.920. At the same time, the frequency of fruit and vegetable consumption, taste preferences, sleep duration, manual labor before retirement, smoking, alcohol consumption, exercise, and social participation were considered.

In terms of interpersonal relationships, we considered the impact of marital status and living arrangements on individual life satisfaction. For living and working conditions, we evaluated two variables: self-assessed economic status and self-assessed health status. The assignment of all variables is shown in Table 1.

**Table 1:** Classification criteria of variables.

Category	Variables	Classification criteria
Individual characteristics	Gender	Female = 0, Male = 1
	Education level	0 year = 0, 0–6 years = 1, >6 years = 2
	Residence	Rural = 0, Urban = 1
	BMI	<18.5 = 0, 18.5–24 = 1, 24–28 = 2, >28 = 3
	Abdominal obesity	No = 0, Yes = 1
	Hypertension	No = 0, Yes = 1
	Heart disease	No = 0, Yes = 1
	Visual impairment	No = 0, Yes = 1
	Hearing impairment	No = 0, Yes = 1
	ADL disability	No = 0, Yes = 1
Psychological and behavioral characteristics	Depressive symptoms	No = 0, Yes = 1
	Anxiety symptoms	No = 0, Yes = 1
	Cognitive impairment	No = 0, Yes = 1
	The frequency of fruit consumption	Never = 0, Occasionally = 1, Often = 2
	The frequency of vegetable consumption	Never = 0, Occasionally = 1, Often = 2
	Dietary preferences	No light = 0, Light = 1
	Sleep duration	<7 h = 0,7–9 h = 1,>9 h = 2
	Smoking	No = 0, Yes = 1
	Drinking	No = 0, Yes = 1
	Manual labor before retirement	No = 0, Yes = 1
	Exercise	No = 0, Yes = 1
	Social participation	No = 0, Yes = 1
Interpersonal relationships characteristics	Marital status	Spinsterhood = 0, Married = 1
	Living arrangements	Living with family = 0, Living alone = 1, Institution = 2
Living and working conditions characteristics	Self-assessed economic status	Good = 0, Fair = 1, Poor = 2
	Self-assessed health status	Good = 0, Fair = 1, Poor = 2

BMI: body mass index, ADL: activities of daily living.

### 2.4 Statistical analysis

The Kolmogorov–Smirnov test is used to assess the normality of continuous variables. Continuous variables that conform to a normal distribution are represented using the mean plus or minus the standard deviation (M ± SD), while categorical variables are described using frequency and percentage (*n*%). In the analysis of differences, t-tests are used for continuous variables that conform to a normal distribution. For categorical variables, chi-square tests are used for analysis. A multivariate Logistic regression model is employed to detect factors influencing life satisfaction in older adults diabetes patients. We assessed potential multicollinearity among predictor variables by calculating Variance Inflation Factors (VIF). Following established methodological guidelines, VIF values below 10 were considered indicative of acceptable levels of multicollinearity (32). Significant influencing factors are further incorporated into a Random Forest model for importance ranking, with the mean decrease in Gini index indicating the importance of variables.

In constructing the random forest model, we optimized the number of trees by monitoring the convergence of the out-of-bag (OOB) error rate (33). The model’s hyperparameters were systematically tuned using a grid search approach, with each parameter combination evaluated through 10-fold cross-validation to identify the optimal configuration. Model performance was comprehensively assessed using multiple metrics, including the area under the receiver operating characteristic curve (AUC), sensitivity, precision, recall, F1 score, and accuracy (34). All analyses were conducted using R 4.3.0. A two-tailed *p*-value less than 0.05 was considered to have significant statistical significance.

## 3 Result

### 3.1 Basic characteristics of the sample

Table 2 describes the individual characteristics of older adults with diabetes. Among the 1,304 participants, 911 (69.86%) were satisfied with their lives, while 393 (30.14%) were not. There were 705 females (54.06%) and 599 males (45.94%). The results of the t-test and chi-square test analyses indicated that there were significant statistical differences in life satisfaction among older adults with diabetes with abdominal obesity (χ^2^ = 15.55, *p* < 0.001) and visual impairment (χ^2^ = 6.48, *p* = 0.011) (Table 2).

**Table 2:** Demographic characteristics and heterogeneity analysis of geriatric diabetes patients in China.

Variables	Total (*n* = 1,304)	Not satisfied (*n* = 393)	Satisfied (*n* = 911)	Statistic	*P*
Age, M ± SD	80.76 ± 10.54	79.93 ± 10.32	81.12 ± 10.61	t = −1.86	0.063
Gender, *n* (%)				χ^2^ = 2.28	0.131
Female	705 (54.06)	200 (50.89)	505 (55.43)		
Male	599 (45.94)	193 (49.11)	406 (44.57)		
Education level, *n* (%)				χ^2^ = 2.37	0.305
0 year	444 (34.05)	136 (34.61)	308 (33.81)		
0–6 years	439 (33.67)	121 (30.79)	318 (34.91)		
>6 years	421 (32.29)	136 (34.61)	285 (31.28)		
Residence, *n* (%)				χ^2^ = 1.79	0.180
Urban	861 (66.03)	270 (68.70)	591 (64.87)		
Rural	443 (33.97)	123 (31.30)	320 (35.13)		
BMI, *n* (%)				χ^2^ = 3.20	0.361
<18.5	98 (7.52)	33 (8.40)	65 (7.14)		
18.5–24	610 (46.78)	192 (48.85)	418 (45.88)		
24–28	451 (34.59)	132 (33.59)	319 (35.02)		
>28	145 (11.12)	36 (9.16)	109 (11.96)		
**Abdominal obesity, *n* (%)**				**χ**^**2**^ **= 15.55**	**<0.001**
**No**	**602 (46.17)**	**214 (54.45)**	**388 (42.59)**		
**Yes**	**702 (53.83)**	**179 (45.55)**	**523 (57.41)**		
**Visual impairment, *n* (%)**				**χ**^**2**^ **= 6.48**	**0.011**
**No**	**1,020 (78.22)**	**290 (73.79)**	**730 (80.13)**		
**Yes**	**284 (21.78)**	**103 (26.21)**	**181 (19.87)**		
Hearing impairment, *n* (%)				χ^2^ = 0.83	0.362
No	906 (69.48)	280 (71.25)	626 (68.72)		
Yes	398 (30.52)	113 (28.75)	285 (31.28)		
Hypertension, *n* (%)				χ^2^ = 0.20	0.652
No	252 (19.33)	73 (18.58)	179 (19.65)		
Yes	1,052 (80.67)	320 (81.42)	732 (80.35)		
Heart disease, *n* (%)				χ^2^ = 0.01	0.941
No	659 (50.54)	198 (50.38)	461 (50.60)		
Yes	645 (49.46)	195 (49.62)	450 (49.40)		
ADL disability, *n* (%)				χ^2^ = 0.02	0.897
No	1,069 (81.98)	323 (82.19)	746 (81.89)		
Yes	235 (18.02)	70 (17.81)	165 (18.11)		

t: t-test, χ^2^: Chi-square test, SD: standard deviation, BMI: body mass index, ADL: activities of daily living. The bold variables indicate that these variables are statistically significant.

Table 3 describes the psychological and behavioral characteristics of older adults with diabetes. The results of the chi-square test analysis indicate that there are significant differences in the frequency of fruit consumption, vegetable consumption, Dietary preferences, sleep duration, drinking, and exercise among older adults with diabetes with different levels of life satisfaction (*p* < 0.05).

**Table 3:** Psychological and behavioral profiling of older adults with diabetes in China: a comparative analysis.

Variables	Total (*n* = 1,304)	Not satisfied (*n* = 393)	Satisfied (*n* = 911)	Statistic	*P*
**Depressive symptoms, *n* (%)**				**χ**^**2**^ **= 63.54**	**<0.001**
**No**	**982 (75.31)**	**239 (60.81)**	**743 (81.56)**		
**Yes**	**322 (24.69)**	**154 (39.19)**	**168 (18.44)**		
**Anxiety symptoms, *n* (%)**				**χ**^**2**^ **= 14.03**	**<0.001**
**No**	**1,187 (91.03)**	**340 (86.51)**	**847 (92.97)**		
**Yes**	**117 (8.97)**	**53 (13.49)**	**64 (7.03)**		
**Cognitive impairment, *n* (%)**				**χ**^**2**^ **= 5.52**	**0.019**
**No**	**1,053 (80.75)**	**302 (76.84)**	**751 (82.44)**		
**Yes**	**251 (19.25)**	**91 (23.16)**	**160 (17.56)**		
**The frequency of fruit consumption, *n* (%)**				**χ**^**2**^ **= 16.46**	**<0.001**
**Never**	**276 (21.17)**	**89 (22.65)**	**187 (20.53)**		
**Occasionally**	**692 (53.07)**	**232 (59.03)**	**460 (50.49)**		
**Often**	**336 (25.77)**	**72 (18.32)**	**264 (28.98)**		
**The frequency of vegetable consumption, *n* (%)**				**χ**^**2**^ **= 12.40**	**0.002**
**Never**	**34 (2.61)**	**12 (3.05)**	**22 (2.41)**		
**Occasionally**	**396 (30.37)**	**145 (36.90)**	**251 (27.55)**		
**Often**	**874 (67.02)**	**236 (60.05)**	**638 (70.03)**		
**Taste preferences, *n* (%)**				**χ**^**2**^ **= 5.67**	**0.017**
**No light**	**404 (30.98)**	**140 (35.62)**	**264 (28.98)**		
**Light**	**900 (69.02)**	**253 (64.38)**	**647 (71.02)**		
**Sleep duration, *n* (%)**				**χ**^**2**^ **= 15.02**	**<0.001**
**<7 h**	**526 (40.34)**	**190 (48.35)**	**336 (36.88)**		
**7–9 h**	**582 (44.63)**	**151 (38.42)**	**431 (47.31)**		
**>9 h**	**196 (15.03)**	**52 (13.23)**	**144 (15.81)**		
Manual labor before retirement, *n* (%)					
No	423(32.44)	131 (33.33)	292 (32.05)	χ^2^ = 0.21	0.650
Yes	881 (67.56)	262 (66.67)	619 (67.95)		
Smoking, *n* (%)				χ^2^ = 2.24	0.135
No	933 (71.55)	270 (68.70)	663 (72.78)		
Yes	371 (28.45)	123 (31.30)	248 (27.22)		
**Drinking, *n* (%)**				**χ**^**2**^ **= 4.35**	**0.037**
**No**	**1,007 (77.22)**	**289 (73.54)**	**718 (78.81)**		
**Yes**	**297 (22.78)**	**104 (26.46)**	**193 (21.19)**		
**Exercise, *n* (%)**				**χ**^**2**^ **= 33.06**	**<0.001**
**No**	**753 (57.75)**	**274 (69.72)**	**479 (52.58)**		
**Yes**	**551 (42.25)**	**119 (30.28)**	**432 (47.42)**		
Social participation, *n* (%)				χ^2^ = 0.00	0.968
No	490 (37.58)	148 (37.66)	342 (37.54)		
Yes	814 (62.42)	245 (62.34)	569 (62.46)		

χ^2^: Chi-square test. The bold variables indicate that these variables are statistically significant.

Table 4 describes the characteristics of interpersonal relationships among older adults with diabetes. Regarding marital status, 47.62% of the patients are unmarried, and 52.38% are married. In terms of living arrangements, 81.67% of the patients live with their families, 15.34% live alone, and 2.99% reside in nursing facilities. The results of the chi-square test analysis indicate that there are significant differences in life satisfaction among older adults with diabetes living in different arrangements (*p* < 0.05).

**Table 4:** Characterization and variability of social networks in Chinese geriatric diabetes population.

Variables	Total (*n* = 1,304)	Not satisfied (*n* = 393)	Satisfied (*n* = 911)	Statistic	*P*
Marital status, *n* (%)				χ^2^ = 0.02	0.889
Marital status, *n* (%)				χ^2^ = 0.02	0.889
Spinsterhood	621 (47.62)	186 (47.33)	435 (47.75)		
Married	683 (52.38)	207 (52.67)	476 (52.25)		
**Living arrangements, *n* (%)**				**χ**^**2**^ **= 6.47**	**0.039**
**Living with family**	**1,065 (81.67)**	**305 (77.61)**	**760 (83.42)**		
**Living alone**	**200 (15.34)**	**75 (19.08)**	**125 (13.72)**		
**Institution**	**39 (2.99)**	**13 (3.31)**	**26 (2.85)**		

χ^2^: Chi-square test. The bold variables indicate that these variables are statistically significant.

Table 5 describes the characteristics of living and working conditions of older adults with diabetes. In terms of self-assessed health status, 38.27% of individuals considered their health to be very good, 43.02% thought their health was average, and 18.71% believed their health was poor. Regarding self-assessed economic status, 22.24% felt their economic situation was very good, 68.40% thought it was average, and 9.36% believed it was poor. The results of the chi-square test analysis indicated that there were significant differences in life satisfaction among older adults with diabetes related to self-assessed of health status (χ^2^ = 189.63, *p* < 0.001) and economic status (χ^2^ = 115.57, *p* < 0.001) (*p* < 0.05).

**Table 5:** Socioeconomic and occupational characterization of older adults with diabetes in China: a comparative analysis.

Variables	Total (*n* = 1,304)	Not satisfied (*n* = 393)	Satisfied (*n* = 911)	Statistic	*P*
**Self-assessed economic status, *n* (%)**				**χ**^**2**^ **= 115.57**	**<0.001**
**Good**	**290 (22.24)**	**33 (8.40)**	**257 (28.21)**		
**Fair**	**892 (68.40)**	**282 (71.76)**	**610 (66.96)**		
**Poor**	**122 (9.36)**	**78 (19.85)**	**44 (4.83)**		
**Self-assessed health status, *n* (%)**				**χ**^**2**^ **= 189.63**	**<0.001**
**Good**	**499 (38.27)**	**46 (11.70)**	**453 (49.73)**		
**Average**	**561 (43.02)**	**214 (54.45)**	**347 (38.09)**		
**Bad**	**244 (18.71)**	**133 (33.84)**	**111 (12.18)**		

χ^2^: Chi-square test. The bold variables indicate that these variables are statistically significant.

### 3.2 Multivariate result analysis

As presented in Table 6, all variance inflation factor (VIF) values were below the threshold of 10, indicating the absence of significant multicollinearity in the logistic regression model. Logistic regression analysis indicates that living arrangements, self-assessment of economic status, self-assessment of health status, exercise, hearing difficulties, anxiety symptoms, and cognitive impairment are significant factors affecting LS among older adults with diabetes (Table 6). The regression coefficient values show that exercise and hearing impairment have a negative effect on life satisfaction of older adults with diabetes and the remaining five significant influences have a positive effect on life satisfaction, which is explained in detail by the OR values. The standard errors of all the variables showed high precision in estimating the regression coefficients. The risk of dissatisfaction with life for patients living alone is 1.59 times that of patients living with family members (OR = 1.59, 95%CI:1.02–2.48), and the risk of dissatisfaction with life for patients living in nursing institutions is 2.53 times that of patients living with family members (OR = 2.53, 95%CI:1.10–5.83). Patients who self-assess their economic status as average have a risk of dissatisfaction with life that is 2.98 times that of patients who self-assess their economic status as good (OR = 2.98, 95%CI:1.93–4.59), and patients who self-assess their economic status as poor have a risk of dissatisfaction with life that is 7.69 times that of patients who self-assess their economic status as good (OR = 7.69, 95%CI: 4.25–13.89). The high OR values indicate that poor self-assessed economic status is a very strong risk factor for life satisfaction in older adults with diabetes. Patients who self-assess their health status as average have a risk of dissatisfaction with life that is 5.03 times that of patients who self-assess their health status as good (OR = 5.03, 95%CI: 3.46–7.31), and patients who self-assess their health status as poor have a risk of dissatisfaction with life that is 9.72 times that of patients who self-assess their health status as good (OR = 9.72, 95%CI: 6.20–15.26). The OR was the highest, indicating that poor self-assessed of health status is also a very strong risk factor for life satisfaction in older adults with diabetes. Patients who exercise have a risk of dissatisfaction with life that is 0.48 times that of patients who do not exercise (OR = 0.48, 95%CI:0.35–0.66), patients with hearing impairment have a risk of dissatisfaction with life that is 0.68 times that of patients without hearing impairment (OR = 0.68, 95%CI:0.48–0.95), patients with depressive symptoms have a risk of dissatisfaction with life that is 1.67 times that of patients without depressive symptoms (OR = 1.67, 95%CI:1.21–2.31), and patients with cognitive impairment have a risk of dissatisfaction with life that is 1.65 times that of patients without cognitive impairment (OR = 1.65, 95%CI:1.12–2.44).

**Table 6:** Determinants of life satisfaction among older adults with diabetes in China: a multivariate logistic regression analysis.

Characteristics	*B*	S.E	*Z*	*P*	OR (95% CI)	VIF
Age	0.00	0.01	0.23	0.822	1.00 (0.98,1.02)	1.514
Gender (female-ref)					1.00	1.379
Male	0.20	0.20	0.99	0.323	1.22 (0.83,1.79)	
Marital status (spinsterhood-ref)					1.00	1.402
Married	0.01	0.20	0.03	0.977	1.01 (0.68,1.49)	
Education level (0 year-ref)					1.00	1.181
0–6 years	−0.05	0.20	−0.28	0.781	0.95 (0.64,1.39)	
>6 years	0.33	0.23	1.44	0.149	1.39 (0.89,2.18)	
Residence (rural-ref)					1.00	1.119
Urban	−0.27	0.17	−1.61	0.107	0.76 (0.55,1.06)	
Living arrangements (living with family-ref)					1.00	1.102
Living alone	0.46	0.23	2.03	**0.043**	**1.59 (1.02,2.48)**	
Institution	0.93	0.43	2.18	**0.029**	**2.53 (1.10,5.83)**	
Self-assessed economic status (good-ref)					1.00	1.052
Average	1.09	0.22	4.93	**<0.001**	**2.98 (1.93,4.59)**	
Bad	2.04	0.30	6.75	**<0.001**	**7.69 (4.25,13.89)**	
Manual labor before retirement (no-ref)					1.00	1.133
Yes	−0.11	0.17	−0.67	0.504	0.89 (0.64,1.24)	
Self-assessed health status (good-ref)					1.00	1.067
Fair	1.62	0.19	8.45	**<0.001**	**5.03 (3.46,7.31)**	
Poor	2.27	0.23	9.90	**<0.001**	**9.72 (6.20,15.26)**	
The frequency of fruit consumption (never-ref)					1.00	1.097
Occasionally	0.12	0.19	0.66	0.510	1.13 (0.78,1.63)	
Often	−0.12	0.23	−0.50	0.617	0.89 (0.57,1.40)	
The frequency of vegetable consumption (never-ref)					1.00	1.080
Occasionally	−0.06	0.46	−0.14	0.889	0.94 (0.38,2.32)	
Often	−0.09	0.45	−0.19	0.851	0.92 (0.38,2.24)	
Taste preferences (no light-ref)					1.00	1.030
Light	−0.04	0.16	−0.28	0.777	0.96 (0.70,1.30)	
Sleep duration (<7 h-ref)					1.00	1.035
7–9 h	−0.11	0.16	−0.71	0.481	0.90 (0.66,1.22)	
>9 h	−0.34	0.22	−1.53	0.127	0.71 (0.46,1.10)	
Smoking (no-ref)					1.00	1.283
Yes	0.04	0.20	0.20	0.844	1.04 (0.71,1.53)	
Drinking (no-ref)					1.00	1.181
Yes	0.37	0.19	1.90	0.057	1.44 (0.99,2.11)	
Exercise (no-ref)					1.00	1.117
Yes	−0.73	0.16	−4.56	**<0.001**	**0.48 (0.35,0.66)**	
Social participation (no-ref)					1.00	1.169
Yes	−0.15	0.17	−0.88	0.380	0.86 (0.62,1.20)	
BMI (<18.5-ref)					1.00	1.075
18.5–24	−0.27	0.29	−0.93	0.352	0.77 (0.44,1.34)	
24–28	−0.36	0.31	−1.15	0.250	0.70 (0.38,1.29)	
>28	−0.53	0.37	−1.41	0.158	0.59 (0.28,1.23)	
Abdominal obesity (no-ref)					1.00	1.168
Yes	−0.24	0.16	−1.47	0.142	0.79 (0.57,1.08)	
Visual impairment (no-ref)					1.00	1.122
Yes	0.08	0.19	0.40	0.690	1.08 (0.75,1.56)	
Hearing impairment (no-ref)					1.00	1.140
Yes	−0.39	0.17	−2.27	**0.023**	**0.68 (0.48,0.95)**	
Hypertension (no-ref)					1.00	1.097
Yes	0.11	0.20	0.58	0.561	1.12 (0.76,1.65)	
Heart disease (no-ref)					1.00	1.093
Yes	−0.06	0.15	−0.40	0.692	0.94 (0.70,1.27)	
ADL disability (no-ref)					1.00	1.181
Yes	−0.32	0.21	−1.51	0.131	0.72 (0.48,1.10)	
Depressive symptoms (no-ref)					1.00	1.101
Yes	0.51	0.16	3.12	**0.002**	**1.67 (1.21,2.31)**	
Anxiety symptoms (no-ref)					1.00	1.081
Yes	−0.12	0.24	−0.48	0.631	0.89 (0.56,1.43)	
Cognitive impairment (no-ref)					1.00	1.134
Yes	0.50	0.20	2.52	**0.012**	**1.65 (1.12,2.44)**	

B: regression coefficients, SE: Standard error, OR: Odds Ratio, CI: Confidence Interval, BMI: body mass index, ADL: activities of daily living. The bold variables indicate that these variables are statistically significant.

### 3.3 Random forest importance assessment

**Figure 2:**
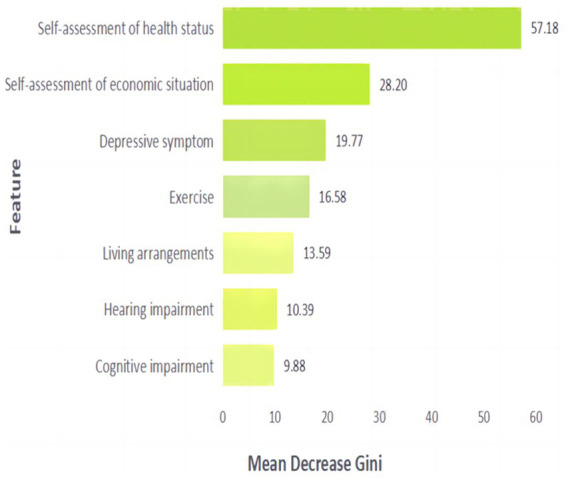
Random forest-based feature importance assessment using mean gini impurity reduction.

Multifactor logistic regression analysis identified seven significant factors, and a random forest model was constructed using these seven significant factors. The AUC of the training set is 0.815 and the values of sensitivity, precision, recall, F1 score and accuracy are 0.948, 0.799, 0.948, 0.867 and 0.797, respectively, while the AUC of the test set is 0.772 and the values of sensitivity, precision, recall, F1 score and accuracy values are 0.883, 0.772, 0.883, 0.824 and 0.736 respectively, which indicates that the performance of random forest model is better. The MDG was used to determine the importance of significant factors on the life satisfaction of patients with diabetes, with a higher MDG indicating that the factor is more important. Figure 2 visualizes the significant factors in the random forest. The most important factor is self-assessed health status, followed by self-assessed economic status, depressive symptoms, exercise, living arrangements, hearing impairment, and cognitive impairment (Figure 2).

## 4 Discussion

Our research results show that 30.14% of older adults patients with diabetes expressed dissatisfaction with their lives. Recent epidemiological studies have reported a 27.5% prevalence of life dissatisfaction among the general older adult population (35). This finding is particularly relevant given that diabetes has been identified as a significant predictor of reduced life satisfaction, as demonstrated in a U.S.-based cohort study (36). Furthermore, emerging evidence suggests that life satisfaction trajectories in older adults individuals with diabetes are mediated by multidimensional factors beyond the disease itself (37). These findings align with our study results, which indicate that life satisfaction in older adults with diabetes is influenced by a complex interplay of physiological, psychological, and social factors associated with disease management.

The self-assessed health status of older adults with diabetes has been identified as a significant factor influencing their LS. A cross-sectional study conducted among older adult populations in northwestern Iran revealed that individuals with prediabetes frequently exhibit negative attitudes toward essential diabetes prevention strategies, including dietary modification, weight management, and physical activity engagement (38). These maladaptive behavioral patterns may contribute to progressive health deterioration and suboptimal disease symptom management, potentially leading to missed opportunities for timely intervention during the critical prediabetes stage (39). Consequently, older adults with diabetes who rate their health as good tend to pay more attention to self-management and health maintenance (40). They are more likely to actively follow medical advice, engage in dietary control, regular exercise, and regular blood glucose monitoring. These healthy behaviors not only help control blood sugar levels but also improve overall health. Furthermore, older adults with diabetes with good self-rated health are more likely to receive social support and recognition (41), which can alleviate psychological stress and enhance their sense of social belonging and well-being. Additionally, patients may feel a sense of pride and satisfaction due to their good health, further boosting their life satisfaction. Good health enables patients to better carry out daily activities and life arrangements (40), enhancing their autonomy and self-efficacy (42), thereby improving life satisfaction.

Economic status has been identified as a significant factor affecting the LS of older adults with diabetes Those with poor economic conditions often face substantial financial pressures (43), such as high medical expenses and the costs of daily diabetes management. A community-based study conducted in rural Crete, Greece, revealed that a significant proportion of patients engage in self-directed medication dose reduction without medical consultation, primarily driven by financial constraints (44). This phenomenon was particularly prevalent among insulin-treated patients, highlighting the socioeconomic challenges in diabetes management. Older adults with diabetes with limited financial resources may also encounter barriers in accessing medical resources and social support (45). The limited medical insurance coverage among migrant workers significantly restricts their access to essential social and policy support systems (46). This systemic barrier undermines the fundamental principles of healthcare accessibility, financial risk protection, and health equity, potentially resulting in suboptimal medical care and limited access to advanced treatment technologies. Consequently, this vulnerable population faces increased risks of treatment delays and disease progression. Furthermore, comparative analyses reveal that migrant workers experience disproportionately higher financial burdens when managing chronic conditions compared to both urban and rural residents with established healthcare access, exacerbated symptoms, and diminished quality of life (47, 48). Earlier research has also demonstrated that individuals with lower incomes often possess a substantially diminished capacity to manage the financial obligations associated with chronic diseases (49). Prolonged financial constraints may be a persistent challenge, with consequent psychological distress and life dissatisfaction. This cumulative effect has been shown to result in a further reduction in life satisfaction. Prolonged uncertainty and financial hardship may also cause individuals to lose confidence in the future, affecting their overall life attitude and well-being (50). Since China’s healthcare reforms, significant investments have been made to strengthen primary healthcare infrastructure and primary healthcare centres have been established in many places (51). However, the inability of primary health care centres to provide individualised services (52) has led to an increase in the number of older adults seeking treatment from secondary or tertiary care institutions. This makes travelling to secondary or tertiary care more costly for the older adults in rural areas where health resources are relatively poor. This suggests that improving access to comprehensive health care at primary health care centres (52) could enable older people to meet their needs through lower costs. This would not only help to reduce their financial burden but also improve their quality of life.

Consistent with other studies, healthy physical conditions are often accompanied by more positive emotional and psychological states (53). Empirical evidence suggests that the presence of positive emotional states serves as both an indicator of superior health status and a protective factor against disease risk (54). When patients experience prolonged depression and anxiety, they may develop mental disorders, which have been confirmed as significant factors affecting life satisfaction (55). Older adults with diabetes have been shown to have a higher risk of developing depression compared to healthy individuals (56). If depression is left undetected, untreated, or inadequately treated, it can impair an individual’s ability to successfully manage diabetes, leading to worsened blood sugar control and a higher risk of complications and adverse outcomes (57). Emerging research evidence highlights the potential protective role of religious belief against depressive disorders (58). The underlying mechanism may involve the mitigation of pessimistic cognitive patterns, thereby enhancing both life satisfaction and psychological well-being (59). These findings align with longitudinal data from the United States, which demonstrated a significantly lower incidence of depression among frequent participants in religious activities during a two-year follow-up period (60). In addition, healthcare providers may consider incorporating routine mental health screening into the management of these patients (61). Timely identification and treatment of depression can improve patients’ quality of life and may increase adherence to diabetes care, thereby further reducing the risk of complications.

The life satisfaction of older adults with diabetes is also influenced by physical activity. Exercise is not only essential for maintaining a healthy physical condition, but also has a positive effect on glycaemic control, improving insulin sensitivity, dyslipidaemia and hypertension (62). It has been demonstrated that regular physical exercise effectively reduces glycaemic levels in patients with diabetes mellitus (63). Furthermore, the combination of progressive resistance training enhances insulin sensitivity in older adults with diabetes and helps to reduce abdominal fat (64). For older adults with diabetes with limited mobility, appropriate exercise can be performed by means of high-intensity interval training characterised by discontinuous, alternating short-term exercises (65), which can be repeated several times. Square dancing, as a popular sport among Chinese middle-aged and older adults people, is often considered to improve the quality of life (66). Research suggests that more than 150 min of moderate to vigorous exercise per week is more effective, and it is optimal to exercise on 2 to 3 non-consecutive days (62). Furthermore, aerobic exercise can alleviate depressive symptoms and improve quality of life (67), positively impacting the mental health of older adults with diabetes, which further enhances life satisfaction.

Older adults with diabetes living alone are more likely to have lower LS than those living with family members. We believe that patients living alone may receive less social support, which diminishes their quality of life (68). In addition, studies have found that patients living alone have poorer dietary habits, including eating fewer types of food and irregular meal times. For the older adults living alone, they cannot adapt to preparing meals for just one person, and a lack of cooking skills may also make it difficult for them to prepare meals (69). The mental health of patients living alone may also affect food intake, leading to increased or decreased food consumption, which further reduces their life satisfaction (70).

Hearing impairment is also a significant factor affecting LS in older adults with diabetes In our study, we observed a phenomenon different from previous research regarding the impact of hearing impairment on life satisfaction in older adults with diabetes Previous studies have indicated that older adults with diabetes with hearing impairment typically have lower life satisfaction compared to those without hearing impairment (71, 72). However, our research results reveal a contrasting outcome: older adults with diabetes with hearing impairment have higher life satisfaction than those without hearing impairment. This phenomenon may be attributed to the widespread adoption of hearing aids and surgical interventions among older adults with diabetes with hearing impairments (73). These rehabilitative measures have effectively mitigated the functional limitations associated with hearing loss, thereby minimizing its impact on daily living activities. After experiencing the dual challenges of hearing impairment and diabetes, older adults with diabetes often reassess their life priorities, adjust their social strategies, and establish high-quality intimate relationships, viewing life with a more peaceful and contented mindset. Although they may objectively face more difficulties and challenges, this change in mindset leads them to subjectively feel better life satisfaction. Additionally, hearing impairment may, to some extent, reduce the interference of external complex social information and social pressure on individuals. With limited hearing, individuals must shift their attention away from a broad social circle and focus more on those close and important to them. Through closer interaction with family members, friends, and other small group members, individuals can establish higher quality intimate relationships. The establishment of these relationships not only provides emotional support and comfort but also enhances the individual’s perception of life satisfaction. This phenomenon may be caused by various factors, including but not limited to differences in research methods. Our study may differ from previous research in sample selection, data collection, and analysis methods, which may have influenced the results. This discrepancy may also stem from response bias during questionnaire completion, where subjective factors or emotional states could compromise the authenticity of self-reported data. Participants may either consciously or unconsciously conceal or exaggerate their actual conditions, potentially affecting the validity of research findings (74). Nevertheless, the precise mechanisms underlying this phenomenon warrant further empirical investigation.

Cognitive impairment can affect memory in the older adults (41), which may lead to patients not taking medication as prescribed, not regularly monitoring their blood sugar, and further deterioration of their condition, thereby reducing their quality of life. Compared to non-patients with diabetes, those with cognitive impairment and diabetes have a shortened lifespan by 4 to 6 years (75), increasing the negative impacts they face. Research has found that good sleep can help overcome negative emotions and enhance happiness (76). Therefore, for older adults with diabetes, optimizing sleep is crucial for alleviating the negative impacts of cognitive impairment and improving quality of life.

The factors affecting life satisfaction of older adults with diabetes revealed in this study have important practical implications in the context of the contemporary Chinese healthcare system. At a time when healthcare payment reform is in full swing and hospital operations are shifting towards refined management, the factors of health self-assessment and depression identified in our study provide ideas for hospitals to optimise chronic disease management pathways. It is recommended that when making a clinical diagnosis, healthcare professionals should not only focus on the patient’s glycaemic control, but also comprehensively assess the patient’s health status and psychological state, so that limited healthcare resources can be accurately invested in high-risk patients through a multifaceted assessment. The significant influence of living arrangement and economic status suggests that the government and society should increase financial support for older adults with diabetes with financial difficulties, and improve their living conditions through the provision of medical assistance and living subsidies. In addition, the government should pay more attention to the mental health and living needs of patients living alone or in institutions, which can be achieved by combining AI algorithms to personalise diabetes management programmes.

## 5 Limitations

This study does have certain limitations. First, the results are derived from the analysis of cross-sectional data, making it difficult to infer causal relationships between variables. Future research should further validate this through longitudinal studies. Second, constrained by the content of the CLHLS database, we used an older dataset, and information on some of the influencing factors that may affect life satisfaction in older adults with diabetes—such as occupation, social policies and potential confounding factors—was not included in this study. In future research, it may be important to understand these potential influencing factors by combining qualitative interview methods. Third, most of the variable data comes from self-reported questions, Participants may be influenced by social expectations when reporting their health status and behaviors, tending to overestimate their health levels or report behaviors that are more in line with social expectations. Self-reported data relies on the participants’ memory, which may lead to recall bias, resulting in inaccurate data. Life satisfaction is self-reported based on a single question, and although previous research has illustrated the reliability of a single question (11), it is still worthwhile to consider more measurement dimensions in future research. Fourth, our study did not explore the interactions between variables in more detail. Future research should focus on the complex relationships between variables to better understand the formation mechanism of life satisfaction among older adults with diabetes. Finally, the patients with diabetes included in the study were not further classified by type. Due to differences in pathogenesis, their lifestyle management may also vary, which should be further subdivided in future research.

## 6 Conclusion

The study further ranked the significance of each significant influencing factor using a random forest model. These findings are of significant guiding importance for the prevention and intervention of low life satisfaction among older adults with diabetes. We advocate that policymakers and relevant health professionals should pay special attention to older adults with diabetes with poor self-assessed health, poor economic conditions, depressive symptoms, short sleep duration, not eating fruit, living alone or in institutions and hearing difficulties when formulating policies and in practical clinical care. The findings of the randomised forest suggest that interventions targeting financial support and whole-cycle health management could be prioritised.

## Data Availability

Publicly available datasets were analyzed in this study. This data can be found at: https://opendata.pku.edu.cn/dataverse/CHADS.
